# Comparative Study of Two Immunisation Protocols in Goats Using Thiol-Sepharose Chromatography-Enriched Extracts from Adult *Haemonchus contortus* Worms

**DOI:** 10.3390/vaccines13070708

**Published:** 2025-06-29

**Authors:** Magnolia M. Conde-Felipe, José Adrián Molina, Antonio Ruiz, Otilia Ferrer, Mª Cristina Del Rio, Emma Carmelo, Juan R. Hernández-Fernaud, Francisco Rodríguez, José Manuel Molina

**Affiliations:** 1 Parasitology Unit, Department of Animal Pathology, Faculty of Veterinary Medicine, University of Las Palmas de Gran Canaria, 35413 Gran Canaria, Spain; magnolia.conde@ulpgc.es (M.M.C.-F.); jose.molina108@alu.ulpgc.es (J.A.M.); otilia.ferrer@ulpgc.es (O.F.); mariacristina.delrio@ulpgc.es (M.C.D.R.); josemanuel.molina@ulpgc.es (J.M.M.); 2 UIC Zoonosis y Enfermedades Emergentes ENZOEM, Universidad de Córdoba, 14071 Córdoba, Spain; 3 Clinical Veterinary Hospital, Faculty of Veterinary Medicine, University of Las Palmas de Gran Canaria, 35413 Gran Canaria, Spain; 4 Institute of Tropical Diseases and Public Health of the Canary Islands, University of La Laguna, 38200 Tenerife, Spain; ecarmelo@ull.edu.es; 5 Department of Obstetrics and Gynecology, Pediatrics, Preventive Medicine and Public Health, Toxicology, Legal and Forensic Medicine and Parasitology, University of La Laguna, 38200 Tenerife, Spain; 6 Department of Biochemistry, Microbiology, Cell Biology and Genetics, University of La Laguna, 38200 Tenerife, Spain; jrfernau@ull.edu.es; 7 Department of Anatomy and Compared Anatomy Pathology, Faculty of Veterinary Medicine, University of Las Palmas de Gran Canaria, 35413 Gran Canaria, Spain; francisco.guisado@ulpgc.es

**Keywords:** *Haemonchus contortus*, immunisation protocol, thiol-binding proteins, goat

## Abstract

**Background**: A comparative analysis was conducted between two immunisation protocols using different amounts of protein extracts from adult *Haemonchus contortus* worms, purified by thiol-Sepharose chromatography (625 μg/animal vs. 200 μg/animal). These protocols involved either five or two inoculations of the immunogen, respectively. **Methods**: To evaluate the level of immunoprotection, animals were challenged with L3 of *H. contortus* two weeks after the last inoculation of the immunogen and humanely sacrificed at 8 weeks post-infection. Parasitological, biopathological, and serological parameters were monitored through the experiment. Parasite burden, abomasal-specific antibody responses, and histopathological changes were determined at the end of the trial. **Results**: The immunisation protocols resulted in similar reductions in cumulative faecal egg counts (60.5–64.9%) and the total worm burden (47.5–50%) compared to non-immunized (control) animals. Overall, these parasitological data showed an early recovery of the haematocrit (PCV) after challenge in the immunised groups relative to control. Similarly, levels of *H. contortus*-specific IgG and IgA antibodies increased in both the serum and gastric mucus of immunised groups. **Conclusions**: These findings represent a further step towards the potential application of this type of immunogen under field conditions, as protective responses (associated with a reduction in faecal egg output) were achieved using a simplified protocol, with lower immunogen doses and fewer inoculations required to induce immunoprotection, thereby mitigating the pathological effects of the parasite and reducing its ability to spread and infect susceptible hosts.

## 1. Introduction

Although gastrointestinal nematodes (GINs) in ruminants do not usually cause very high mortality rates, these parasitic infections lead to significant economic losses due to reduced growth rates, decreased carcass quality, diminished milk production, and impaired reproductive performance. Even subclinical infections can increase an animal’s susceptibility to other pathogens and consistently have a negative impact on animal welfare [1,2].

Among the alternative strategies to the use of anthelmintics for the control of GINs in ruminants, the development of vaccines is being actively explored [3]. Many studies have demonstrated that parasitic antigens of various types can induce a degree of immunoprotection, although not to the extent achieved against less complex organisms such as protozoa [4]. Consequently, it appears unlikely that helminth vaccines will provide full protective immunity. Instead, the aim is to stimulate a host immune response that limits the number of worms completing their endogenous cycle, thereby mitigating the adverse effects on animal health and productivity and/or reducing transmission to other susceptible hosts [5].

Strategies for vaccination against GINs have included the use of different types of native (somatic or from excretion/secretion (E/S) products) [6,7] and, more recently, recombinant immunogens [8,9,10]. This range of vaccine candidates has been extended by using different methods of antigen presentation, including the use of different adjuvants [11,12], routes of administration, and DNA-based vaccines [13,14], among others. These factors have been associated with highly variable results in the level of immunoprotection achieved. In the case of immunization strategies against *H. contortus*, a vaccine based on immunisation with hidden parasite antigens (H-gal-GP and H11) is currently on the market. This vaccine has produced very good results in sheep [15] but has sometimes shown less effectiveness in goats [16]. These differences have been attributed to different evolutionary strategies or to differences in the mechanisms involved in post-vaccination responses in both species [17].

In the context of vaccine development against GINs, our research group has conducted several immunisation trials in small ruminants using thiol-sepharose chromatography-enriched protein fractions (TBSP) obtained from adult *Haemonchus contortus* (*H. contortus*) worms [18]. These studies showed that these immunogens are able to induce significant protection against the parasite, as evidenced by reductions in adult worm burden and faecal egg count (FEC) following challenge with infective L3 of the parasite. This protective response was associated with a Th2-type humoral immune response, which is already present in immunised animals during the prepatency period [19,20].

All the previous studies using thiol-binding extracts from adult *H. contortus*, employed protocols involving up to five inoculations administrated at weekly intervals, requiring large quantities of immunogen. The present study aimed to evaluate simplified immunization protocols. In this context, we assessed the level of protection through parasitological and biopathological analyses in goats immunised with *H. contortus* adult worm fractions (TBSP) using simplified protocols (fewer inoculations and lower concentration of immunogen) compared to previous studies.

## 2. Materials and Methods

### 2.1. Parasites and Immunogens

A strain of *Haemonchus contortus* originally isolated from naturally infected goats in Gran Canaria (Spain) was maintained in goats for this study. Protein extracts used as immunogens were prepared following established protocols [19,20]. Adult worms were processed to obtain soluble proteins, which were purified through buffer exchange and affinity chromatography using Thiol-Sepharose (Amersham Pharmacia Biotech, Uppsala, Sweden). Proteins eluted with buffer containing 25 mM L-cysteine were desalted again and referred to as TBSP fractions.

### 2.2. Immunization Trials

Twenty-one healthy 9-month-old male goats (Majorera breed) were reared in covered pens under conditions designed to preclude helminth infections. The animals were divided into the following weight-balanced groups:

- Group 1 (*n* = 6): goats immunized with a total of 625 μg TBSP in Freund complete and incomplete adjuvants and challenged with *H. contortus*; immunization in this group of animals was performed by weekly intramuscular injections of 50, 75, 100, 100, and 300 μg of TBSP extract over five consecutive weeks. Freund complete adjuvant was used for the first immunization (50 μg), and Freund incomplete adjuvant for the following immunizations. This protocol has previously been used in several experiments in goats demonstrating an immunoprotective effect against *H. contortus.* [19,20].
- Group 2 (*n* = 6): goats immunized with a total of 200 μg TBSP in Freund complete and incomplete adjuvants and challenged with *H. contortus*; immunization in this group of animals was performed by weekly intramuscular injections of 100 μg TBSP extract over two consecutive weeks. Freund complete adjuvant was used for the first immunization and Freund incomplete adjuvant for the second.
- Group 3 (*n* = 6): Freund adjuvant control. Animals in control group 3 underwent the same inoculation protocol as animals in group 1, but elution buffer was used instead of PBS-TSBP.
- Group 4 (*n* = 3): This group contained uninfected and unimmunised animals and served as a control for serological analysis.

To evaluate the level of immunoprotection, animals from groups 1, 2, and 3 were challenged with 7000 L3 of *H. contortus* two weeks after the last inoculation of the immunogen and humanely sacrificed at 8 weeks post-infection. The L3 used in the experimental infections were obtained by coproculture (14 days at 25 °C) of faeces from goats monospecifically infected with *H. contortus.* Larvae were recovered using the modified Baermann technique following the protocol proposed by [21]. Experimental infections were carried out by intraruminal puncture.

### 2.3. Haematological and Parasitological Analysis

Eggs per gram (EPG) were determined by a modified McMaster technique. To determine the number of adult worms, after opening the abomasum of the slaughtered animals and the collection of mucus samples from the gastric fundus (see Section 2.4), the abomasums were washed with dH_2_O. A 200 mL aliquot of the total volume of liquid obtained from each animal after abomasal washing was preserved in 5% formalin. These samples were used for worm counts, which were then extrapolated to estimate the total parasite burden based on the full volume recovered from each animal. Larval burden was determined by digestion of mucosal scrapings with pepsin-HCl and expressed as the number of immature worms per gram of mucosa [22].

For haematological determinations, blood samples were collected in test tubes containing EDTA. Packed cell volume (PCV) was determined using a microhaematocrit, and plasma protein (PP) was estimated using a refractometer (Comecta S.A.). The results for both parameters are expressed in % and g/dL, respectively.

### 2.4. ELISA

Indirect ELISA was used, as previously described [19] to determine the presence of specific antibodies against *H. contortus* adult worm fractions (TBSP) in serum samples. ELISA plates were coated with TBSP at a concentration of 5 micrograms/mL at 4 °C overnight and serum samples were analysed at a dilution of 1/200 or 1/50 for IgG or IgA determinations, respectively. Conjugates (Bio-Rad Laboratories, Inc. Hercules, CA, USA) were employed at a dilution of 1/6000 (anti-goat IgG-peroxidase) or 1/1500 (anti-goat IgA-peroxidase). Results were expressed as relative units according to the optical density (OD) observed in a positive sample (a pool of five serum samples from goats that were immunised with the same immunogen -TBSP from adult *H. contortus* worms- in previous studies, which showed high levels of specific antibodies). Serum samples from animals in group 4 were used as negative controls.

To assess specific mucosal IgG and IgA levels, abomasal mucus was collected by superficial scraping of the mucosal surface. The samples were diluted at a ratio of 2.5 mL buffer (0.1 M Na_2_HPO_4_, 0.05 M NaCl, 3 mM NaN_3_, 1 mM PMSF [Sigma-Aldrich Corp., St. Louis, MO, USA], and 5 mM EDTA; pH 7.1) per gram of mucus, homogenised, and centrifuged at 18,000× *g* for 30 min. Supernatants were stored at −20 °C prior to ELISA analysis. Optimal assay parameters were defined using pools of known positive and negative mucus samples. TBSP concentrations for ELISA coating were 3.0 µg/mL for IgG and 10.0 µg/mL for IgA detection. Mucus samples were diluted in PBS at 1:20 for IgG and 1:5 for IgA. Detection employed peroxidase-conjugated anti-goat IgG (Bio-Rad Lab. Inc. USA) at 1:1500 and anti-goat IgA (Acris GmbH, Heidelberg, Germany) at 1:1000, both diluted in PBS.

A citric acid-phosphate buffer containing 0.04% (*w*/*v*) o-phenylenediamine dihydrochloride (OPD) and 0.1% (*v*/*v*) H_2_O_2_ was used as substrate. All samples were analyzed in duplicate, and the optical densities (OD) were determined at a wavelength of 492 nm (Multiskan Ascent 354, Thermo Labsystems Inc., Pittsburgh, PA, USA) [19,20].

### 2.5. Histology

Histological analysis was performed on tissue samples from the abomasal mucosa, which were cut into 5 μm thick sections and stained with Giemsa and haematoxylin-eosin. The number of eosinophils, globule leukocytes, mast cells, and plasma cells were determined. The counts of these cell subsets were conducted at 400× magnification in 40 randomly selected fields of 0.038 mm^2^, located in the upper and lower thirds of the mucosa. The results were expressed as cells/mm^2^ [19]. Histological evaluations were performed in a blinded manner to reduce observational bias.

### 2.6. Statistical Analysis

All statistical analyses were performed using IBM SPSS Statistics for Windows, version 22.0 (IBM Corp., Armonk, NY, USA). For comparisons between groups at individual time points, the non-parametric Mann–Whitney U test was applied. For variables measured weekly throughout the study (e.g., serum IgA and IgG concentrations, faecal egg count [FEC], packed cell volume [PCV], and pepsinogen levels) were analysed using a repeated-measures General Linear Model (GLM), following assessment of data distribution with the Shapiro–Wilk test. Data not normally distributed were square root transformed before GLM analysis. The Wilcoxon signed-rank test was also employed to compare the effect of the challenge on matched samples (from the same experimental group) throughout the study. This test was used to analyse the evolution of parameters in each group such as PCV and PP levels in samples from immunised (G1 and G2) and control (G3) groups. Finally, the Spearman correlation test was used to analyse the association between the various parameters evaluated in the study. *p* < 0.05 was considered statistically significant.

## 3. Results

### 3.1. Parasitological and Haematological Results

**Figure 1:**
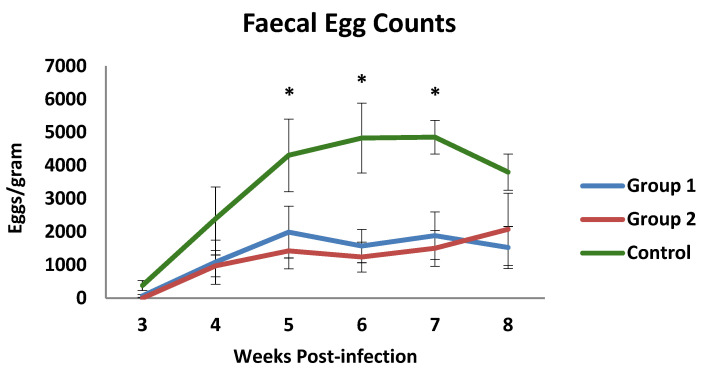
Faecal egg counts in groups immunized with TBSP from adult *H. contortus* worms (groups 1 and 2) and in control animals (group 3) after challenge with 7000 infective L3 larvae of the parasite. Results are mean values of eggs per gram of faeces ± SEM * *p* < 0.05.

Coproscopical data are presented in Figure 1 as faecal egg counts (FECs) (mean value of eggs per gram of faeces ± SEM-EPG). The prepatent period was found to be 3 weeks in both the control and the immunised groups. Throughout the experiment, the mean counts of both immunised groups showed mean EPG values that were lower than those observed in the control group, particularly on week 7 p.i. At this time, faecal counts of the control group reached the highest mean value (4855 EPG). The mean EPG values observed in both immunized animals were lower than in the control group from week 4 p.i., showing significant differences when they were compared at weeks 5, 6, and 7 p.i. Consequently, the cumulative faecal counts showed lower mean values in the immunised animals with a 60.5% (group 1) and a 64.9% (group 2) reduction in cumulative counts at the end of the study.

**Figure 2:**
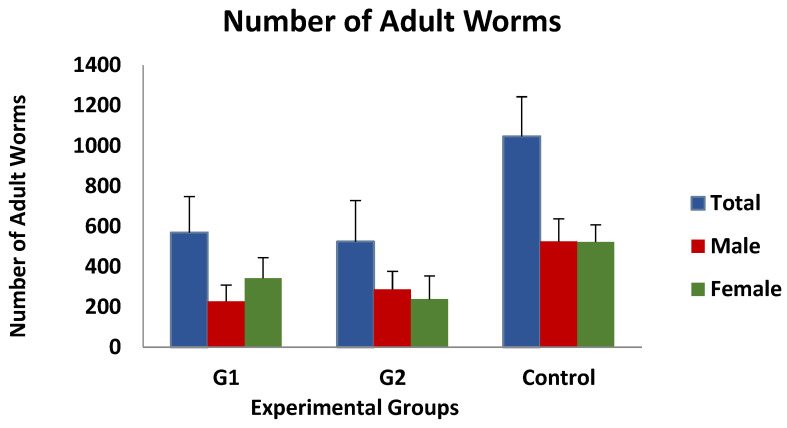
Mean adult worm counts (total, females and male worms) in groups immunized with TBSP from adult *H. contortus* worms (groups 1 and 2) and in control animals (group 3) after challenge with 7000 infective L3 larvae of the parasite. Results are mean ± SEM groups.

Immature worm counts observed after digestion of the gastric mucosa were found to be very low, with mean values of approximately 0.5 larvae/gram of mucosa, and no differences were observed between the three groups investigated. In contrast, as shown in Figure 2, the mean value of adult worm counts in the immunised groups was 568 ± 178 (group 1) and 524 ± 203 (group 2), while the mean number of worms detected in the control group was 1046 ± 196 worms/animal. According to the data presented, the immunised animals exhibited a 45.7% (group 1) or 50% (group 2) reduction in the total number of adult worms compared to the control group, although these differences were not statistically significant. Moreover, the analysis of the data according to the sex of the adult worms revealed no significant differences.

**Figure 3:**
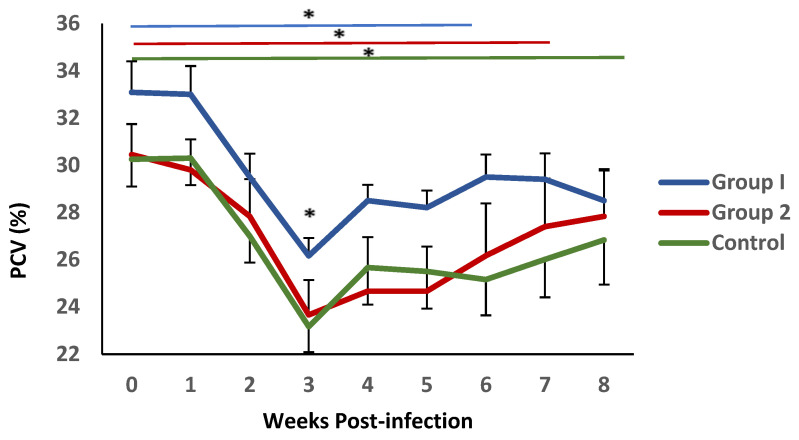
Evolution of packed cell volume (PCV) in groups immunized with TBSP from adult *H. contortus* worms (groups 1 and 2) and in control animals (group 3) after challenge with 7000 infective L3 larvae of the parasite. Results are mean percentage (%) values ± SEM * *p* < 0.05.

The mean values of PCV levels in blood samples from all experimental groups are shown in Figure 3. Following challenge, a significant decrease in this parameter was observed during the first three weeks after infection., reaching minimum values at 3 weeks p.i., coinciding with the conclusion of the pre-patent period. This reduction was more pronounced in group 2 and the control animals. Significant differences were observed between the mean PVC values observed in group 1 and the control group at this time point. This parameter demonstrated a gradual recovery; nevertheless, it failed to reach the initial values. During this period, the recovery process appeared to be more pronounced in group 1, where no statistical differences with the initial values were detected from week 5 post-infection. In contrast, in group 2 and the control, this recovery was not observed until weeks 6 and 7 post-infection, respectively.

**Figure 4:**
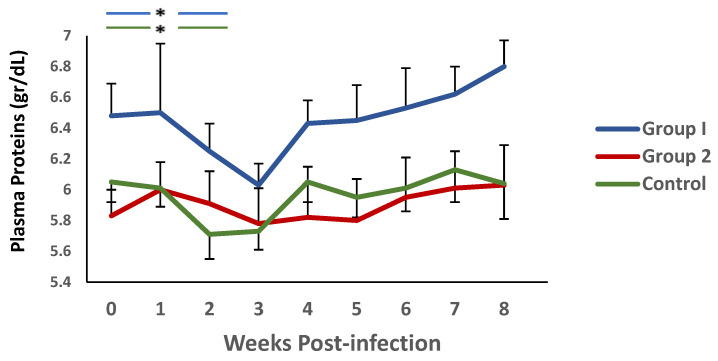
Evolution of plasma proteins (gr/dL) levels in groups immunized with TBSP from adult *H. contortus* worms (groups 1 and 2) and in control animals (group 3) after challenge with 7000 infective L3 larvae of the parasite. Results are mean ± SEM * *p* < 0.05.

A similar trend was observed when plasma protein levels were analysed (Figure 4). Again, there was a decrease in this parameter during the first 3 weeks p.i., and an increase thereafter. It is noteworthy that significant differences were observed between group 1 and the control during weeks 4 and 5 p.i.

On the other hand, when analysing the evolution of this parameter in each of the groups, in relation to the values observed at the time of challenge, using the Wilcoxon signed rank test, no significant differences were observed in group 2. However, both group 1 and the controls showed a significant reduction in PP levels during the first two weeks p.i. in relation to the values found in each group at the time of the challenge.

Regarding the statistical associations between the biopathological and parasitological data, negative correlations were always observed between both types of data. Thus, at the end of the study, both cumulative faecal counts and number of worms showed a r = −0.680 (*p* < 0.05) and r = −0.703 (*p* < 0.05), respectively, in relation to PCV. The same parasitological parameters were also negatively associated with PP levels (cumulative faecal counts: r = −0.466, *p* = 0.052) (number of worms: r = −0.558; *p* < 0.05).

### 3.2. ELISA Tests

**Figure 5:**
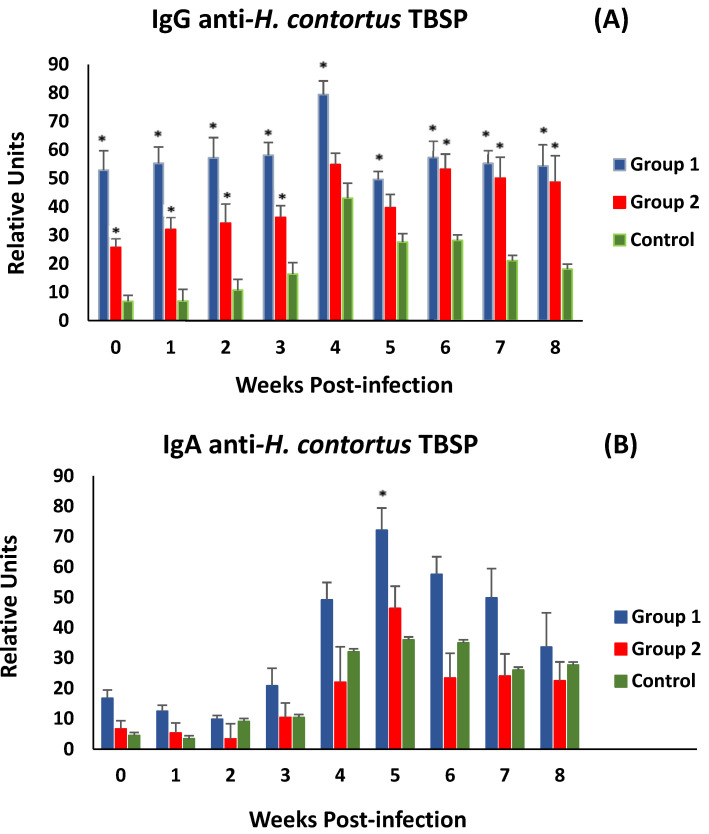
Evolution of levels of specific IgGs (**A**) and IgAs (**B**) in serum against thiol-binding somatic proteins (TBSP) fractions from *H. contortus* in groups immunized with TBSP from adult *H. contortus* worms (groups 1 and 2) and in control animals (group 3) after challenge with 7000 infective L3 larvae of the parasite. Results are mean ± SEM * *p* < 0.05.

Figure 5A shows the evolution of anti-*H. contortus* TBSP IgG levels in serum samples after challenge (week 0 p.i.). After the challenge, both immunised groups did not show significant differences in anti-TBSP IgG levels until the end of the study. Both groups of immunised animals (G1 and G2) showed significantly higher levels of specific antibodies at the time of experimental infection than those observed in the control group. After experimental infection, animals in all experimental groups showed a response with an increase in specific antibody levels, peaking at week 4 p.i. However, immunised animals showed higher levels of specific IgG throughout the study, with significant differences from the control group (except in group 2, at weeks 4 and 5 p.i.).

Analyses of associations between specific IgG levels and various parasitological (worms and faecal egg count) and biopathological (PCV and PP) parameters were negative for parasitological data and positive for biopathological data, but no statistical significance was found. A similar trend was observed when serum anti-TBSP IgA levels were analysed. However, in this case, only animals immunised from G1 showed higher mean levels than the control group throughout the study. Significant differences were observed at 5 weeks post-challenge (Figure 5B). On the other hand, the same type of association was observed as in the specific IgG analyses, positive for PCV and PP values and negative for parasitological data.

**Figure 6:**
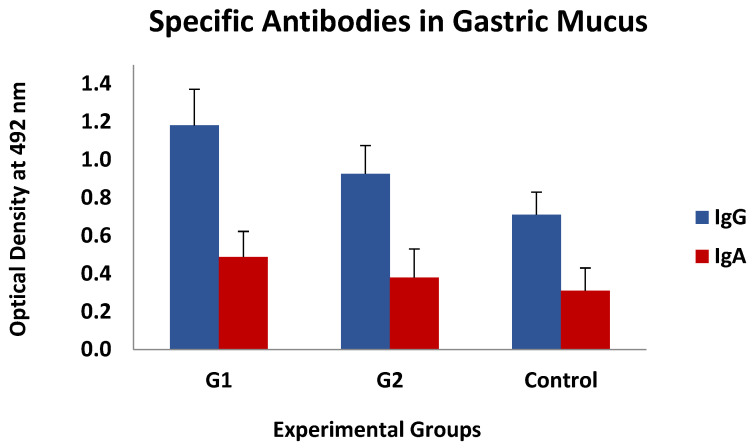
Levels of specific IgGs and IgAs in gastric mucus against thiol-binding somatic proteins (TBSP) fractions from *H. contortus* in groups immunized with TBSP from adult *H. contortus* worms (groups 1 and 2) and in control animals (control, group 3) after challenge with 7000 infective L3 larvae of the parasite at the end of the study. Results are mean ± SEM.

This type of association was also found, again without statistical significance, between the levels of specific IgGs in the mucus, whereas they could not be detected when analysing the IgA response. The mean values of both specific antibody isotypes (IgG and IgA) observed in gastric mucus are shown in Figure 6, which demonstrates that the immunised animals had higher mean values than the control ones, especially in G1, but these differences were not statistically significant.

### 3.3. Histology

**Figure 7:**
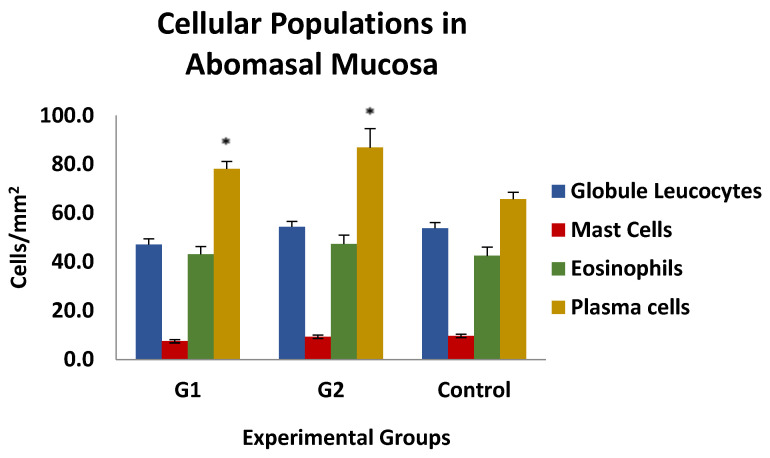
Level of cellular populations in the gastric mucosa in groups immunized with TBSP from adult *H. contortus* worms (groups 1 and 2) and in control animals (control, group 3) after challenge with 7000 infective L3 larvae of the parasite. Results are mean number of cells/mm^2^ ± SEM. * *p* < 0.05.

The results of the histological examination of the gastric mucosa of the animals in the different experimental groups are shown in Figure 7. No differences were observed between the different cell populations, except for plasma cells, which were significantly increased in both immunised groups (G1 and G2) compared to the control group. No association was found between the number of different cell populations analysed and the parasitological, biopathological, and serological parameters studied, except for globule leukocytes, whose presence in mucosal inflammatory infiltrates in both immunised groups was negatively and significantly associated with PCV and PP levels at weeks 3 and 6 post-infection, with r-values ranging from −0.489 and −0.543 or −0.403 and −0.643 for PCV and PP, respectively.

## 4. Discussion

The aim of the present study was to evaluate the efficacy of two immunisation protocols using thiol-sepharose chromatography-enriched protein fractions (TBSP) from adult *H. contortus* worms in goats. The results showed that both protocols, even when using a lower immunogen dose and number of inoculations (200 µg/2 inoculations in group 2 vs. 650 µg/5 inoculations in group 1), conferred protection against the parasite. This was evidenced by reductions in faecal egg count (FEC) and adult worm burden. These findings are consistent with previous trials demonstrating the potential of TBSP extracts to induce protective immune responses in small ruminants [18,19,20,23].

The reduction in FEC observed in both immunised groups (60.5% in group 1 and 64.9% in group 2) is comparable to the levels of protection reported previously using similar immunogens [18,19,20,23] and can be considered relevant when compared to the numerous studies that have evaluated the immunoprotective effect of different native and recombinant *H. contortus* proteins/extracts [3,17,24]. The reduction in faecal egg counts after immunisation observed in the present study suggests that the simplified protocol (lower immunogen dose and lower number of inoculations), could offer a practical and field-applicable strategy to significantly reduce environmental egg shedding, a crucial factor in controlling the spread of *H. contortus*, as it directly limits the number of infective larvae available to infect other animals. Similarly, the faecal egg count results are consistent with the adult worm burden results (47.5% in group 1 and 50% in group 2), further supporting the efficacy of both immunisation protocols tested. This concordance indicates a direct impact on the parasite’s ability to establish and/or persist in the host, reinforcing the conclusion that both protocols provide similar levels of immunoprotection.

Haematological analysis revealed a decrease in PCV and PP values following challenge with *H. contortus* L3 larvae, a typical response to this infection due to blood and protein loss in the host’s abomasum [25]. However, PCV levels gradually recovered in vaccinated animals. By week 6 post-infection (group 1) and week 7 (group 2), PCV values were not significantly different from those recorded at the time of challenge. In contrast, mean PCV levels in the control group remained below baseline. These findings suggest that vaccination helps to reduce parasite burden and mitigate some of the physiological damage caused by the infection, supporting the consistency of the parasitological results.

Although biopathological responses varied considerably in the goat breed used in this study [26], the overall recovery of PCV levels was more evident in vaccinated animals compared to controls, consistent with observations from previous vaccination trials against this parasite [27]. This improvement was more pronounced in group 1; however, no significant differences were observed between the two vaccinated groups in the evaluated parameters. These results suggest that the simplified immunisation protocol provides comparable protection to the five-dose regimen against the parasite’s pathogenic effects, resulting in faster PCV recovery in both cases.

All these parasitological and haematological findings were accompanied by increased levels of specific antibodies (IgG and IgA) in the serum and gastric mucosa of the immunised groups compared with the control group. As observed in previous studies, the development of these serological responses suggests that the extracts used do not contain hidden antigens, based on the secondary antibody response detected at the serum level following challenge. This aligns with previous findings indicating that Th2-mediated humoral immune responses play a key role in protective immunity, either in naturally resistant sheep breeds [28] or experimentally induced by TBSP extracts in goats [19,20]. The higher levels of specific antibodies in group 1, particularly at the time of challenge, may explain the more pronounced recovery of PCV levels in this group. However, the absence of a significant correlation between antibody levels and parasitological parameters suggests that additional immune mechanisms may also contribute to the protection [8,11,29] or that the results are a consequence of the variability observed in these parameters within the different experimental groups [12].

Histopathological analysis revealed an increase in plasma cell infiltration in the gastric mucosa of immunized animals, indicating a local immune response. This finding is in accordance with previous studies that have highlighted the importance of mucosal immunity in controlling gastrointestinal nematode infections [30]. The presence of plasma cells in the mucosa may contribute to the production of local antibodies that could play a role in limiting parasite establishment and/or fecundity, although in this case no clear relationship could be established between the type of cells infiltrating the mucosa and the parasitological, biopathological, or serological findings. With regard to globule leukocytes, although vaccinated animals did not show higher mean values than the control ones, it was the only effector cell population that could be associated with increased resilience to vaccination-induced infection, as shown by the negative correlations with PCV and PP levels in the vaccinated groups, supporting the idea that these cells could play an important role against this parasite [19,30].

## 5. Conclusions

This study demonstrates that immunisation with TBSP extracts of adult *H. contortus* worms can induce a significant reduction in faecal egg counts in infected goats, even when using simplified protocols involving fewer inoculations (2 vs. 5) and lower doses of immunogen (200 µg/animal vs. 625 µg/animal). Immunisations also resulted in a reduction in adult worm counts that does not reach statistical significance, which could be determined by the variability observed when analysing this parameter and the sample size. This reduction in parasite load that could contribute to the observation of lower cumulative faecal egg counts, together with the improvement in the recovery of some haematic parameters such as PCV in vaccinated animals, support the potential of these immunisation protocols as a possible strategy to control *H. contortus* infections in goats. However, further research is required to optimise immunization protocols and assess the benefits of combining TBSP extracts with other immunogens or adjuvants. A limitation of this study is the use of controlled experimental conditions, which may not fully reflect field environments. Therefore, additional studies under natural infection conditions and in larger animal populations are essential to evaluate the practical efficacy and scalability of these vaccines.

## Data Availability

Data are contained within the article.
